# Anti-inflammatory T-cell shift in neuropathic pain

**DOI:** 10.1186/s12974-014-0225-0

**Published:** 2015-01-21

**Authors:** Benjamin Luchting, Banafscheh Rachinger-Adam, Jens Heyn, Ludwig Christian Hinske, Simone Kreth, Shahnaz Christina Azad

**Affiliations:** Department of Anesthesiology and Pain Medicine, Ludwig-Maximilians University Munich, Marchioninistr. 15, 81377 Munich, Germany

**Keywords:** Neuropathic pain, T-cells, TH17, Treg, Cytokines, Neuroinflammation

## Abstract

**Background:**

The classification of pain into nociceptive and neuropathic pain is based on characteristic symptoms and different pathophysiological mechanisms. In a recent investigation, we found a disrupted TH17/Treg balance in patients suffering from chronic unspecific low back pain (CLBP). These patients did not show any signs of neuropathy. There is evidence for a considerable impact of the immune system also in neuropathic pain. However, the role of the adaptive immune system is still unclear. In the present study, we investigated systemic T-cell subset responses and T-cell related cytokine profiles in patients with chronic neuropathic pain.

**Methods:**

We analyzed T-cell subsets, mRNA expression and T-cell-related cytokine profiles in 26 patients suffering from neuropathic pain in comparison to 26 healthy controls. Using multicolor flow cytometry (FACS), we quantified the number of T helper cells 1 (TH1), TH2, TH17 and regulatory T-cells (Tregs). Forkhead-Box-Protein 3 (FoxP3), Transforming growth factor-β (TGF-β) and RAR-related orphan receptor-γT (ROR-γT) mRNA expression was determined by quantitative real-time PCR (qPCR) and levels of pain-related cytokines were measured by Human Cytokine Multiplex Immunoassay (Macrophage inflammatory protein-1α (MIP-1α), Tumor necrosis factor-α (TNF-α), Interferon-γ (IFN-γ), Interleukin (IL) -4, IL-6, IL-10, IL-17, and IL-23).

**Results:**

We found a TH17/Treg imbalance with significantly increased anti-inflammatory Tregs and decreased pro-inflammatory TH17 cells in patients with neuropathic pain as compared to healthy controls. These results were confirmed on mRNA level: Treg-related FoxP3 and TGF-β mRNA expression was elevated, whereas expression of TH17-related RORγT was reduced. Cytokine analyses revealed only marginal changes.

**Conclusions:**

Our investigation revealed a clear shift of T-cell subsets towards anti-inflammation in patients with neuropathic pain. Interestingly, this is quite similar to our previous findings in CLBP patients, but even more pronounced. Therefore, it remains to be elucidated in future investigations whether the immune changes represent an underlying pathophysiological mechanism or an epiphenomenon induced by ongoing pain and stress.

**German Clinical Trial Register (DRKS):**

Trial registration number: DRKS00005954

**Electronic supplementary material:**

The online version of this article (doi:10.1186/s12974-014-0225-0) contains supplementary material, which is available to authorized users.

## Background

Neuropathic pain represents a major problem in clinical practice. In contrast to nociceptive pain, which is caused by damage or potential damage to tissue, neuropathic pain occurs due to a lesion or disease of the peripheral or central nervous system. It is characterized by burning and lancinating pain sensations and further somatosensory disturbances like hypo- and hypersensitivity. Very often, neuropathic pain is more difficult to treat and more refractory to common analgesics, including non-steroidal anti-inflammatory drugs and opioids, than nociceptive pain [1,2]. Despite extensive research, the underlying pathophysiological mechanisms of neuropathic pain are still not fully understood. In recent years, increasing evidence indicates a pivotal role of the immune system in neuropathic pain [3,4]. The majority of previously published data link pain syndromes with higher levels of pro-inflammatory cytokines. Due to these findings, attempts were made in numerous studies to reduce neuropathic pain by blocking pro-inflammatory or enhancing anti-inflammatory immune cells and cytokines [3]. For example, in animal models of neuropathy, tumor necrosis factor-α (TNF-α), Interleukin (IL) -6, IL-17 and Makrophage inflammatory protein1-α (MIP1-α) blockers reduced pain hypersensitivity [5-9]. Comparable results were obtained by increasing the anti-inflammatory cytokines IL-4, IL-10 or Transforming growth factor-β (TGF-β) [10-12]. Nevertheless, despite those promising experimental findings, there are no pharmacological agents available for the specific immunological therapy of neuropathic pain until now.

Cytokines and neutrophils are important during the early stages of acute pain, whereas T-lymphocytes seem to play a central role in chronic neuropathic pain [13]. Regarding T-cells as key players of the adaptive immune system, a TH1/TH2 imbalance has already been shown in patients with complex regional pain syndrome (CRPS) and chronic pelvic pain [14,15]. In recent years, TH1/TH2 dichotomy has been extended by the identification of two other CD4^+^ T-cell lineages: TH17 and regulatory T-cells (Tregs) [16]. TH17 cells appear to be the key effector T-cells in a variety of human autoimmune diseases and Tregs play a vital role in controlling adaptive immune responses. In neuropathy, TH17 has been linked to increased pain sensitivity and destructive effects promoting persistent pain [16], while Tregs were found to be mainly involved in the endogenous recovery [17]. Recently published data showed an increased proportion of Tregs in patients with postherpetic neuralgia [18]. Assuming a beneficial role for an anti-inflammatory T-cell shift, a phase one trial was carried out with an anti-CD28 antibody, preferentially expanding TH2 cells and Tregs in human volunteers. Despite promising results in several animal models, the clinical trial had to be cancelled because of severe side effects [19]. In patients with neuropathic pain, the role of T-cell subsets has not yet been investigated.

In a recent study, we found that patients suffering from nociceptive, non-specific chronic low back pain (CLBP), without any signs of neuropathic pain components, display a clear disruption of the TH17/Treg balance as compared to healthy volunteers [20]. Since clinical and pathophysiological mechanisms differ considerably between nociceptive and neuropathic pain, we aimed to detail changes of pro- and anti-inflammatory T-cell subsets and the respective relative mRNA expression, as well as pain-related cytokine levels in patients with chronic neuropathic pain in comparison to pain free controls. While the cytokine measurement did not reveal any relevant results, we found a distinct anti-inflammatory shift of the T-cell subsets and their respective mRNA expression.

## Methods

### Ethics statement

The study followed the principles of the Declaration of Helsinki and was approved by the Ethics Committee of the Ludwig Maximilians University Munich (Ethical approval number: 331–10). This study was registered on German Clinical Trial Register (Trial registration: DRKS00005954).

### Subjects

Patient recruitment of our prospective study was estimated to last for two years. All patients presented to our Department of Anesthesiology and Pain Medicine, Ludwig-Maximilians University Munich with neuropathic pain for at least six months were assessed for fulfillment of the inclusion criteria and asked for their consent to participate in the study. In addition, healthy pain-free volunteers without any signs or history of pain were asked for their participation. Neuropathic pain was diagnosed according to its international definition: ‘pain caused by a lesion or disease of the somatosensory nervous system’ [2], by pain history, physical examination and the PainDETECT questionnaire.

This questionnaire consists of several items related to neuropathic symptoms (burning sensations, tingling or prickling sensations, shooting or lancinating, hyperalgesia, dysesthesia, allodynia or paresthesia) with excellent sensitivity (85%) and specificity (80%) [21]. Additionally, quantitative sensory testing (QST) was performed in all patients according to the protocol of the German Research Group on neuropathic pain [22]. Patients with mixed pain (nociceptive and neuropathic components) like complex regional pain syndrome (CRPS) and low back pain with radiculopathy were excluded. Further exclusion criteria were autoimmune, chronic systemic, inflammatory, neoplastic or psychiatric diseases, as well as drug abuse and pregnancy. Patients taking any current medication with opioids, non-opioids or co-analgesics were excluded. None of the patients had been treated with corticosteroids or had received immunomodulatory agents currently or in the past. Any signs of acute inflammatory disease were disclosed by laboratory examination, including plasma concentration of C-reactive protein (CRP), total and differential leucocyte count, as well as measurement of the body temperature. Patients rated their recalled average pain intensity using an 11-point numerical rating scale (NRS): 0 meaning ‘no pain’ and 10 meaning ‘worst pain imaginable’.

Self-perceived stress was evaluated using the German version of the Questionnaire for Actual Demands (KAB: *Kurzfragebogen zur aktuellen Beanspruchung*) in patients and healthy volunteers. The KAB was designed to repeatedly quantify an individual’s acute or chronic stress. It is highly sensitive to short-term or situational changes during a stressful experience. The rating is based on a six-point scale ranging from one to six based on normalized adjectives. Higher KAB values indicate increased perceived levels of stress [23].

### Cytokine assessment

Samples of peripheral blood from all patients and healthy controls were collected between 9:00 and 9:30 am, centrifuged at 2000 × g/10 min and stored in polypropylene aliquot tubes at −80°C. Samples were then assessed for levels of T-cell-related cytokines using a human cytokine multiplex immunoassay (Myriad Rules-Based Medicine Inc., Austin, Texas, United States). The multiplex microbead assay is based on Luminex technology and measures proteins in a similar manner to standard sandwich ELISA, with comparable sensitivity and range. Regarding the detection limits, the lower limit of quantitation (LLOQ) for the cytokines were: MiP1-α: 42.0 pg/ml, TNF-α: 23.0 pg/ml, IFN-γ: 1.5 pg/ml, IL-4: 29.0 pg/ml, IL-6: 11.0 pg/ml, IL-10: 6.9 pg/ml, IL-17: 4.0 pg/ml, and IL-23: 0.59 pg/ml. The LLOQ is the lowest concentration of an analyte in a sample that can be reliably detected and at which the total error meets the laboratory’s requirements for accuracy [24].

### Flow cytometric staining and analysis

**Figure 1:**
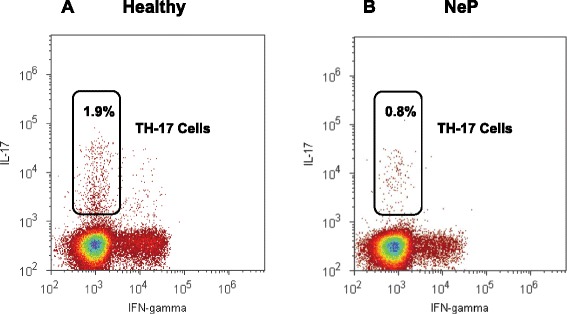
**Gating strategy for the detection of pro-inflammatory TH17 cells.** PBMCs were stained with Brilliant Violet (BV421)-labeled anti-human IL-17 antibody. Lymphocyte population was gated from PBMCs according to forward scatter (FSC) characteristics and side scatter (SSC) characteristics (see Figure 2) and then separated in IL-17 ^+^ TH17 cells. Representative results of a healthy control with a higher number of TH17 cells **(A)** and a patient suffering from neuropathic pain with less TH17 cells **(B)** are shown. TH, T helper cells; PBMC, Peripheral blood mononuclear cells; IL, Interleukin.

**Figure 2:**
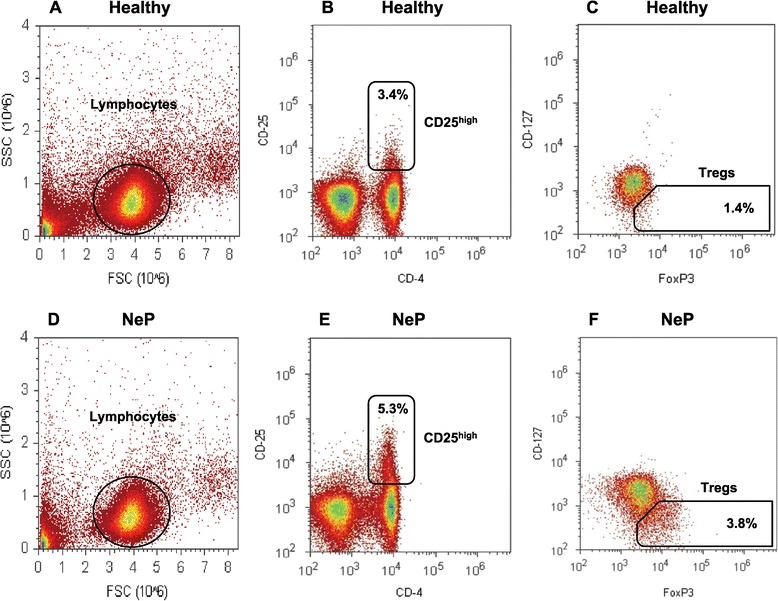
**Gating strategy for the detection of the investigated Tregs.** PBMCs were extracellularly stained with PerCP-labeled anti-human CD4-, PE-labeled anti-CD25 antibody, Brilliant Violet (BV570)-labeled anti-CD127 antibody and intracellularly stained with Alexa Fluor (AF488)-labeled anti-human FoxP3 antibody. Lymphocyte population was gated according to forward scatter (FSC) characteristics and side scatter (SSC) characteristics (A + D). Gated lymphocytes were then separated in CD4 ^+^ CD25^high^ cells (B + E) and CD4 ^+^ CD25^high^CD127^low^FoxP3^+^ cells (C + F, named Treg). Representative results of a healthy control **(A-C)** and a patient with neuropathic pain **(D-F)** are shown. Regulatory T-cells, Tregs; PBMC, Peripheral blood mononuclear cells; Forkhead-Box-Protein 3, FoxP3.

Peripheral blood mononuclear cells (PBMCs) were separated by density gradient preparation over Ficoll-Uropoline (Sigma Aldrich, Taufkirchen, Germany) of all heparinized venous blood samples. Then, PBMCs were cryopreserved in Roswell Park Memorial Institute medium (RPMI) freezing media (Sigma Aldrich, Taufkirchen, Germany), containing 10% Fetal calf serum (FCS), (Sigma Aldrich, Taufkirchen, Germany) and 10% Dimethyl sulfoxide (DMSO), (Sigma Aldrich, Taufkirchen, Germany) [25], and stored at −30°C for 24 hours, and then at −196°C until measurement. After storage, samples were thawed rapidly and washed twice to eliminate DMSO. For TH1, TH2 and TH17 analysis, cells were stimulated for five hours with cell stimulation cocktail, including protein transport inhibitors Phorbol 12-myristate 13-acetate (PMA), ionomycin, Brefeldin A and monensin (eBioscience, San Diego, California, United States), according to the manufacturer’s protocol. Subsequently, cells were extracellularly stained with anti-human CD4 antibody and consecutively fixed and permeabilized (Fix-Perm-Solutions A and B, Life Technologies, Darmstadt, Germany) for intracellular staining with anti-human Interferon-γ, Interleukin (IL) -4 and IL-17 antibody (Biolegend, San Diego, California, United States). T-cell distribution was measured by fluorescent-activated cell sorting (FACS) analysis with the Attune Acoustic Focusing Cytometer (Life Technologies, Carlsbad, United States), and exemplary pictures of the gating strategy for TH17 cells are displayed in Figure 1 and Additional files 1 and 2. Tregs were identified and quantified after surface staining of PBMCs with monoclonal antibodies (mAbs) specific for anti-human CD4, CD25 and CD127 and intracellular staining with an anti-human FoxP3 antibody (Biolegend, San Diego, California, United States). The frequencies of CD4^+^CD25^high^ T-cells and CD4^+^CD25^high^CD127^low^FoxP3^+^ T-cells were expressed as percentage of total CD4^+^ T-cells by sequential gating on lymphocytes. Exemplary pictures of the gating strategy for Tregs are displayed in Figure 2 and Additional files 1 and 2. Isotype controls (Biolegend, San Diego, California, United States) were given for compensation and confirmation of antibody specificity.

### Quantitative real-time PCR (qPCR)

CD4^+^ cells were isolated from PBMCs by magnetic separation with Whole Blood CD4 MicroBeads (MACS Miltenyi Biotec, Auburn, California, United States) according to the manufacturer’s recommendations. Subsequently, total RNA was isolated using the mirVana miRNA Isolation Kit followed by a DNase-digest with Turbo DNA-free Kit (Ambion, Darmstadt, Germany). Quantity and purity of the isolated RNA were measured using a NanoDrop ND-1000 spectrophotometer (Peqlab, Erlangen, Germany). After amplification of total RNA using TargetAmp 1-Round aRNA Amplification Kit (Epicentre Biotechnologies, Madison, Wisconsin, United States) and purification using RNeasy Mini Kit (Qiagen, Hilden, Germany), cDNA synthesis was performed with SuperScript III First Strand Synthesis System (Invitrogen, Darmstadt, Germany) and random hexamers (Qiagen, Hilden, Germany). Quantitative RT-PCR was performed in duplicates with the LightCycler 480 instrument (Roche Diagnostics, Mannheim, Germany) using LightCycler 480 Probes Master and RealTime ready single assays (Roche Diagnostics, Mannheim, Germany) and UniversalProbeLibrary (UPL) probes. The RealTime ready single assays contain target-specific primers and a UPL-LNA probe (Roche Diagnostics, Mannheim, Germany). Primer sequences and qPCR characteristics are given in Table 1. The cycling conditions comprised an initial denaturation phase at 95°C for 10 minutes, followed by 45 cycles at 95°C for 10 seconds, 60°C for 30 seconds and 72°C for one second. Relative mRNA expression of FoxP3, TGF-β and RORγT was calculated by Relative Quantification Software (Roche Diagnostics, Mannheim, Germany) using an efficiency-corrected algorithm with standard curves and reference gene normalization against the reference genes succinate dehydrogenase complex subunit A (SDHA) and TATA box binding protein (TBP) as described in Ledderose *et al.* [26].

**Table 1:** **RT-PCR assay characteristics and primer sequences**

**Gene**	**Primer sequence**
SDHA	Roche RealTime Ready Single Assay ID 102136
TBP	Roche RealTime Ready Single Assay ID 101145
FoxP3	Roche RealTime Ready Single Assay ID 113503
TGF-β	for 5′ACTACTACGCCAAGGAGGTCAC 3′
	rev 5′TGCTTGAACTTGTCATAGATTTCG 3′, UPL probe #31
RORγT	for 5′CAGCGCTCCAACATCTTCT 3′
	rev 5′CCACATCTCCCACATGGAC 3′, UPL probe #69

### Statistical analyses

Statistical analyses were performed using SigmaStat 12.0 (Systat Software, Chicago, United States). Every statistical analysis was started with testing for normal distribution using the Shapiro-Wilk test. Testing for differences between groups was accomplished by the *T*-test for all data with normal distribution and the nonparametric Mann–Whitney rank sum test for all data without normal distribution. Family-wise error rate was controlled at a false discovery level of q <0.05, and *P* values were adjusted accordingly following the Benjamini-Hochberg algorithm. *P* <0.05 were considered to be statistically significant. Results are expressed as mean ± standard deviation (SD) in the text.

## Results

### Subjects

Within two years of recruitment, 26 patients fulfilling the inclusion criteria and 26 healthy controls were enrolled. The characteristics of the participating patients and pain syndromes are given in Table 2.

**Table 2:** **Patient characteristics**

**Item**	**Healthy**	**NeP (all)**	**PNeP**	**PHN**	**OFP**
**Numbers (n)**	26	26	13	7	6
**Age**	39 ± 11	56 ± 14*	49 ± 14*	71 ± 6*	53 ± 10*
**Female**	52%	73%	69%	71%	83%
**BMI**	23.4 ± 2.8	24.0 ± 3.4	25.1 ± 3.2	22.4 ± 3.4	23.6 ± 3.8
**NRS (rest)**	0.0 ± 0.0	4.5 ± 2.3*	4.3 ± 2.5*	3.7 ± 1.6*	5.6 ± 2.1*
**NRS (motion)**	0.0 ± 0.0	6.2 ± 2.7*	6.0 ± 2.3*	4.7 ± 3.9*	6.6 ± 3.3*
**KAB**	1.9 ± 0.7	3.2 ± 0.8*	3.3 ± 0.7*	2.9 ± 1.1*	3.1 ± 0.8*

NeP (all): all patients suffering from neuropathic pain; PNeP: Peripheral neuropathic pain (symmetrical polyneuropathy/peripheral mononeuropathy); PHN: postherpetic neuralgia; OFP: orofacial pain; NRS (rest/motion): Numeric rating scale (0 to 10) of pain, 0: ‘no pain’, 10: ‘worst pain imaginable’; KAB: Questionnaire for self-perceived stress ranging from one (no stress) to six (maximum stress). Results are expressed as mean ± standard deviation (SD), *P <0.05 versus healthy controls.

### Granulocytes and lymphocytes were only slightly changed in neuropathic pain

Venous blood was drawn between 9:00 and 9:30 am into vacutainers containing Ethylenediaminetetraacetic acid (EDTA) for routine laboratory studies. Upon analyzing the number of neutrophil granulocytes, representing an essential part of the innate immune system, as well as lymphocytes, we found only slight alterations in patients with neuropathic pain (neutrophils: 55.4 ± 9.1% in controls versus 58.6 ± 9.3% in neuropathic pain, *P* = 0.268; lymphocytes: 33.8 ± 8.1% in controls versus 29.2 ± 8.2% in neuropathic pain, *P* = 0.069, Table 3).

**Table 3:** **Differential blood count, flow cytometric and RT-PCR results of patient subgroups**

**Item**	**Healthy**	**NeP (all)**	**PNeP**	**PHN**	**OFP**
Neutrophils (%)	55.4 ± 9.1	58.6 ± 9.3	57.5 ± 10.1	57.3 ± 9.0	62.3 ± 8.5
Lymphocytes (%)	33.8 ± 8.1	29.2 ± 8.2	30.5 ± 7.9	28.3 ± 9.6	27.2 ± 8.1
CD4^+^ Counts × 1000	28.7 ± 7.3	33.5 ± 15.4	36.8 ± 18.8	30.3 ± 15.3	30.8 ± 2.9
CD4^+^ (%)	43.4 ± 9.9	47.5 ± 11.9	49.9 ± 12.6	44.3 ± 13.8	46.6 ± 8.0
TH1 (%)	9.7 ± 4.7	9.6 ± 4.1	10.9 ± 4.7	7.2 ± 3.1	9.3 ± 2.7
TH2 (%)	1.3 ± 1.2	1.5 ± 0.8	1.7 ± 0.7	0.9 ± 0.5	2.0 ± 0.9
TH17 (%)	1.3 ± 1.0	0.7 ± 0.4*	0.9 ± 0.4	0.4 ± 0.1*	0.8 ± 0.5
RORγT	2.7 ± 1.4	1.9 ± 1.0	1.8 ± 0.6*	1.9 ± 1.2*	2.2 ± 1.5
CD4^+^CD25^high^ (%)	3.7 ± 0.7	5.4 ± 1.5*	5.1 ± 1.8*	5.0 ± 0.7*	6.4 ± 0.9*
Tregs (%)	2.0 ± 1.0	3.9 ± 1.3*	3.5 ± 1.1*	4.0 ± 1.9*	4.4 ± 0.9*
FoxP3	0.6 ± 0.2	1.2 ± 0.8*	1.0 ± 0.7*	1.3 ± 0.3*	1.4 ± 1.3*

NeP (all): all patients suffering from neuropathic pain; PNeP: Peripheral neuropathic pain (symmetrical polyneuropathy/peripheral mononeuropathy); PHN: postherpetic neuralgia; OFP: orofacial pain; Results are expressed as mean ± standard deviation (SD), *P <0.05 versus healthy controls.

### Cytokine measurement did not reveal relevant results

The specific functions of T-cell subsets are based on their respective cytokine release. TH1 cells produce predominantly pro-inflammatory cytokines such as IFN-γ and TNF-α, which support cellular immunity, whereas TH2 cells release anti-inflammatory cytokines, including IL-4 and IL-10, which mediate humoral immunity. TH17 cells particularly produce the potent pro-inflammatory cytokine IL-17, which is involved in many inflammatory conditions. IL-23 is a key cytokine in the control of inflammation in peripheral tissues, which stimulates naïve CD4 T-cells to differentiate into TH17 cells, in conjunction with IL-6 and TGF-β. Tregs have an anti-inflammatory role by releasing anti-inflammatory cytokines like IL-10 and TGF-β. However, it seems likely, that TH1 and TH17 cytokines are central to increased pain sensitivity, whereas TH2 and Treg derived cytokines may be protective.

Therefore, we analyzed blood levels of pain-associated and T-cell-related cytokines using human cytokine multiplex immunoassay (MIP-1α, TNF-α, IFN-γ, IL-4, IL-6, IL-10, IL-17, and IL-23). Serum levels of IL-4, TNF-α and IFN-γ were neither detectable in the peripheral blood of patients nor in healthy controls. No differences between patients and healthy controls were found analyzing IL-6, IL-10 and IL-17. In accordance with numerous described studies, serum levels of pro-inflammatory cytokines MIP-1α and IL-23 were significantly higher in the peripheral blood of patients suffering from neuropathic pain. However, it has to be noted that IL-23 was the only cytokine with values above the so-called LLOQ, the lowest concentration that can be reliably detected (see Methods section). (IL-6: 1.2 ± 0.8 pg/ml in controls versus 2.5 ± 2.4 pg/ml in neuropathic pain, *P* = 0.064; IL-10: 3.56 ± 2.45 pg/ml in controls versus 3.69 ± 2.40 pg/ml in neuropathic pain, *P* = 0.84; IL-17: 3.53 ± 2.11 pg/ml in controls versus 4.29 ± 2.02 pg/ml in neuropathic pain, *P* = 0.23; MIP-1α: 17.2 ± 11.2 pg/ml in controls versus 28.4 ± 16.4 pg/ml in neuropathic pain, *P* = 0.022; IL-23: 0.9 ± 0.3 pg/ml in controls versus 1.2 ± 0.4 pg/ml in neuropathic pain, *P* = 0.022).

### TH17 frequency was distinctly decreased in neuropathic pain

**Figure 3:**
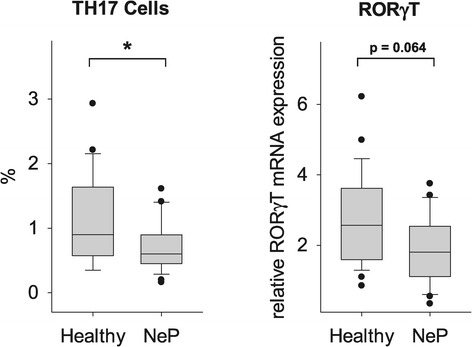
**Reduced TH17 frequency in patients with neuropathic pain.** Using a multicolor flow cytometer, PBMCs were intracellularly stained with anti-human IL-17 antibody after five hours of stimulation. The results show a significantly decreased frequency of TH17 cells in the peripheral blood of patients suffering from neuropathic pain. In accordance with these results, the relative mRNA expression of the TH17 cell-specific transcription factor RORγT was reduced, but did not reach significance. T helper cells, TH; Peripheral blood mononuclear cells, PBMC; Interleukin, IL; RAR-related orphan receptor-γT, ROR-γT; *P <0.05 versus healthy controls.

Although many studies have analyzed the role of TH17 cells in human autoimmune diseases, there are very limited data on the role of TH17 cells in patients with neuropathic pain. TH17 cells act as an important pro-inflammatory component and have been shown to promote inflammation in a number of diseases. The proportion of TH17 cells is expressed as percentage of all T-cells. As shown in Figure 3, the frequency of TH17 cells was evidently decreased in the peripheral blood of patients suffering from neuropathic pain. Affirming these results, the relative mRNA expression of the TH17 cell-specific transcription factor RORγT was reduced, but did not reach significance (TH17 cells: 1.3 ± 1.0% in controls versus 0.7 ± 0.4% in neuropathic pain, *P* = 0.046; relative RORγT mRNA expression: 2.7 ± 1.4 in controls versus 1.9 ± 1.0 in neuropathic pain, *P* = 0.064; Figure 3).

### Treg frequency was distinctly increased in neuropathic pain

**Figure 4:**
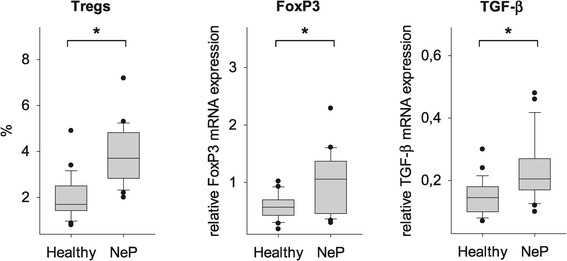
**Increased Treg frequency in patients with neuropathic pain.** In addition to the TH17 cell quantification, the number of Tregs was analyzed after intra- and extracellular staining procedure. CD4 ^+^ CD25^high^CD127^low^FoxP3^+^ were defined as Tregs. The results show a significantly increased frequency of Tregs in patients with neuropathic pain as compared to healthy controls. To confirm the quantitative observations of the Treg frequency, the relative mRNA expression of the Treg-specific transcription factor FoxP3 as well as TGF-β was determined by quantitative real-time PCR (qPCR). Affirmatively, increased mRNA levels of FoxP3 and TGF-β were consistent with the flow cytometric analyses. Regulatory T-cell, Treg; T helper cell, TH; Forkhead-box-protein 3, FoxP3; Transforming growth factor-β, TGF-β; *P <0.05 versus healthy controls.

Human regulatory T-cells play a vital role in controlling the adaptive immune response and in maintaining self-tolerance. Tregs have been shown to prevent autoimmune diseases and to limit chronic inflammatory and nervous system disturbances. On the other hand, the strong Treg-induced immune suppression also impairs beneficial responses such as anti-tumor immunity [27,28]. However, there were limited data analyzing the functional role of Tregs in neuropathic pain, which therefore remained to be investigated. We analyzed the number of Tregs by flow cytometry, using two staining procedures; classic extracellular staining with CD4^+^CD25^high^ and the more specific intra- and extracellular staining procedure with CD4^+^CD25^high^CD127^low^FoxP3^+^. We defined CD4^+^CD25^high^CD127^low^FoxP3^+^ T-cells as Tregs. The prevalence of Tregs was expressed as a ratio of CD4^+^CD25^high^CD127^low^FoxP3^+^ T cells as a percentage of CD4^+^ T-cells. Figure 3 shows a significantly increased frequency of Tregs in patients with neuropathic pain as compared to controls. To confirm the quantitative observations of the Treg frequency we also determined the relative mRNA expression of the specific transcription factor FoxP3 and TGF-β by quantitative real-time PCR (qPCR). As shown on Figure 4, significantly increased mRNA levels of FoxP3 and TGF-β were observed in patients with neuropathic pain. These results were consistent with the flow cytometric analyses (Tregs: 2.0 ± 1.0% in controls versus 3.9 ± 1.3% in neuropathic pain, *P* = 0.007; relative FoxP3 mRNA expression: 0.6 ± 0.2 in controls versus 1.2 ± 0.8 in neuropathic pain, *P* = 0.028; relative TGF-β mRNA expression: 0.15 ± 0.06 in controls versus 0.25 ± 0.15 in neuropathic pain, *P* = 0.009; Figure 4).

### TH1/TH2 balance was only slightly altered in neuropathic pain

**Figure 5:**
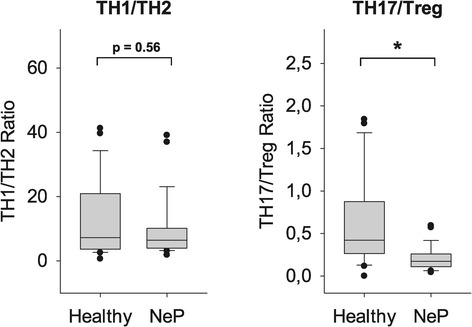
**Altered TH1/TH2 and TH17/Treg balances in patients with neuropathic pain.** While the TH1/TH2 ratio was only slightly decreased, the TH17/Treg balance was significantly enhanced in patients as compared to healthy controls. These results indicate a clear anti-inflammatory T-cell shift in neuropathic pain. T helper cell, TH; Regulatory T-cell, Treg; *P <0.05 versus healthy controls.

In previous investigations, the ratio of TH1 and TH2 cells was used to characterize immune responses in different diseases. In the present study, a trend towards a decreased TH1/TH2 ratio was observed, which, however, did not reach significance (TH1/TH2: 16.1 ± 17.4 in controls versus 10.1 ± 10.0 in neuropathic pain, *P* = 0.56; Figure 5).

### TH17/Treg balance was markedly disrupted in neuropathic pain

TH17 cells play an important pro-inflammatory role whereas Tregs are strong immune suppressors. Therefore, the balance between TH17 cells and Tregs, along with TH1/TH2 balance, is an important factor in analyzing the immune response. Our results regarding T-cell subsets alone demonstrated markedly reduced pro-inflammatory TH17 cells with simultaneous elevated anti-inflammatory Tregs. Conclusively, as shown in Figure 5, the TH17/Treg ratio was significantly lower in the peripheral blood of patients compared to healthy controls. These results indicate a clear anti-inflammatory T-cell shift in neuropathic pain (TH17/Treg: 0.9 ± 1.1 in controls versus 0.2 ± 0.1 in neuropathic pain, *P* <0.007; Figure 5).

## Discussion

Neuropathic pain is a severe and frequent condition which affects up to 18% of the population [29]. The pathophysiological mechanisms leading to chronification of neuropathic pain are a major focus of interest, but are not yet completely elucidated. Recent data indicate a critical involvement of the innate and adaptive immune system in the pathophysiology of chronification. Several types of immune cells have been implicated in the pathogenesis of neuropathic pain [3]. The innate immune system has been shown to be important during the early stages of acute pain, represented particularly by neutrophils [13]. Regarding chronification, T-lymphocytes, as key players of the adaptive immune system, seem to be of major importance [30,31]. Traditionally, it has been suggested that neuropathic pain is associated with a pro-inflammatory immune response. Therefore, mainly anti-inflammatory treatments targeting cytokines and immune cells have been evaluated in several animal models of neuropathic pain [3]. In a recent study, neuropathic pain induced by experimental autoimmune neuritis was successfully attenuated by expanding Tregs [17]. In humans, the first Treg-expanding tests were stopped because of life-threatening side effects [19]. Nevertheless, the modulation of T-cells is still the focus of intense research [32].

In the present study, we analyzed the pain-related cytokines MIP-1α, TNF-α, IFN-γ, IL-4, IL-6, IL-10, IL-17 and IL-23 in the peripheral blood of 26 patients with neuropathic pain and compared the results with those of 26 healthy controls. We found that the serum levels of IL-4, TNF-α and IFN-γ were below the detectable limit and no differences were found regarding IL-6, IL-10 and IL-17. Only the pro-inflammatory cytokines MIP-1α and IL-23 were significantly higher in neuropathic pain. It has to be noted though, that except for IL-23, values of all cytokines measured were below the lowest concentration of an analyte in a sample that can be reliably detected. This shortcoming of serum cytokine measurements in pain syndromes has also been described by other authors [33]. Taken together, our results indicated that serum cytokine levels alone are not sufficient to monitor the adaptive immune response in neuropathic pain and led us to analyze the cellular compartment.

By routine laboratory studies regarding the differential leucocyte count, we found only an unchanged number of neutrophil granulocytes and a slightly reduced number of lymphocytes in the peripheral blood of patients with neuropathic pain. Using multicolor flow cytometry, we subsequently quantified the numbers of TH1, TH2, TH17 and Treg cells in the peripheral blood of our patients. Contrary to our initial assumption, we found clear indications for an anti-inflammatory T-cell shift: Pro-inflammatory TH17 cells were significantly decreased, whereas anti-inflammatory Tregs were significantly increased. Consequently, the corresponding TH17/Treg ratio was distinctly shifted towards an anti-inflammatory immune response. To confirm the quantitative observations of the TH17 and Treg frequency, we also determined the relative mRNA expression of the TH17 cell-specific transcription factor RORγT, as well as TGF-β and the Treg-specific transcription factor FoxP3 by quantitative real-time PCR. A diminished RORγT mRNA expression was in line with the reduced number of TH17 cells, while a notably elevated FoxP3 and TGF-β mRNA expression confirmed the increased Treg frequency. Regarding patient subgroups, we found no differences in respect of the anti-inflammatory T-cell shift and mRNA expressions between patients suffering from orofacial pain, postherpetic neuralgia and other types of peripheral neuropathies (Table 3). The question arises whether the observed changes are of clinical relevance, particularly in view of the overall low portion of the specific T-cell subsets. Furthermore, it would be interesting and relevant to investigate whether the immune changes can also be found in the affected tissue.

Our results are, at first glance, unexpected since the majority of previously published data describes the association between pain and ‘immune activation’ based on investigations of TH1 and TH2 cells, as well as cytokines. This previous TH1/TH2 paradigm has, however, been revised and updated with the discovery of TH17 cells and the more specific detection of Tregs. Our findings, together with recently published data regarding various T-cell subsets, point to a strong association between pain and ‘immune suppression’. Interestingly, the T-cell response in the present study is comparable with our recent findings in patients with CLBP, who also presented with high pain and stress levels, but had no signs of neuropathic pain [20]. There is a general consensus that neuropathic and nociceptive pain are distinct entities, although basic research clearly reveals a huge overlap of underlying pathophysiological mechanisms, including neurotransmitters and cytokines [2]. Our results show for the first time that in both neuropathic and nociceptive pain the adaptive immune system is altered in the same anti-inflammatory way. The context of chronic stress and immune suppression has been described for many years, although not extensively with regard to TH17 cells and Tregs [34]. An anti-inflammatory T-cell shift has been found in patients with chronic mild depression or chronic fatigue syndrome [35-37], and both disorders are frequently associated with all types of chronic pain.

Concerning the cellular mechanisms, T-cell differentiation mainly depends on the cytokine milieu of the microenvironment, but other pathways have also been shown to be involved. For example, the hypothalamic-pituitary-adrenal axis mediates immune regulation through binding of stress hormones like adrenocorticotropic hormone or cortisol to their cognate receptors at the surface of T-cells. Furthermore, the sympathetic nervous system is known to induce immune dysregulation via adrenaline and noradrenaline [38]. These processes in turn play an important role in negative emotional states, such as stress and depression. Our patients with neuropathic pain also suffered from stress and psychological burden as revealed by the enhanced KAB values. We therefore hypothesize that the altered immune responses in both of our studies might reflect a particular chronic pain-related stress reaction

## Conclusions

In summary, we found a TH17/Treg imbalance with increased anti-inflammatory Tregs and decreased pro-inflammatory TH17 cells in patients with neuropathic pain. These results are quite similar to our previous findings in patients with nociceptive CLBP who did not show any signs of neuropathy, but similar pain and stress levels. Therefore, it remains to be clarified in future studies whether the immune changes represent an underlying pathophysiological mechanism or an epiphenomenon induced by ongoing pain and stress.

## Additional files

Additional file 1: Figure S1. Exemplary density plots of 10 healthy controls and 10 patients with neuropathic pain showing pro-inflammatory TH17 cells. Additional file 2: Figure S2. Exemplary density plots of 10 healthy controls and 10 patients with neuropathic pain showing anti-inflammatory Tregs.

## Abbreviations

CLBP: Chronic low back pain
FACS: Fluorescent-activated cell sorting
RT-PCR: Quantitative real-time PCR (qPCR)
TH: T-helper cell
Treg: Regulatory T-cell
FoxP3: Forkhead-Box-Protein P3
TGF-β: Transforming growth factor-β
RORγT: RAR-related orphan receptorγT
MIP1-α: Macrophage inflammatory protein1-α
TNF-α: Tumor necrosis factor-α
IFN: Interferon
IL: Interleukin
KAB: German version of the Questionnaire for Actual Demands “*Kurzfragebogen zur aktuellen Beanspruchung*”
