# Supplementary figures and images for: Anti-inflammatory T-cell shift in neuropathic pain
Source: J Neuroinflammation. 2015 Jan 21;12:12. doi: 10.1186/s12974-014-0225-0 (PMC4308022; doi:10.1186/s12974-014-0225-0)

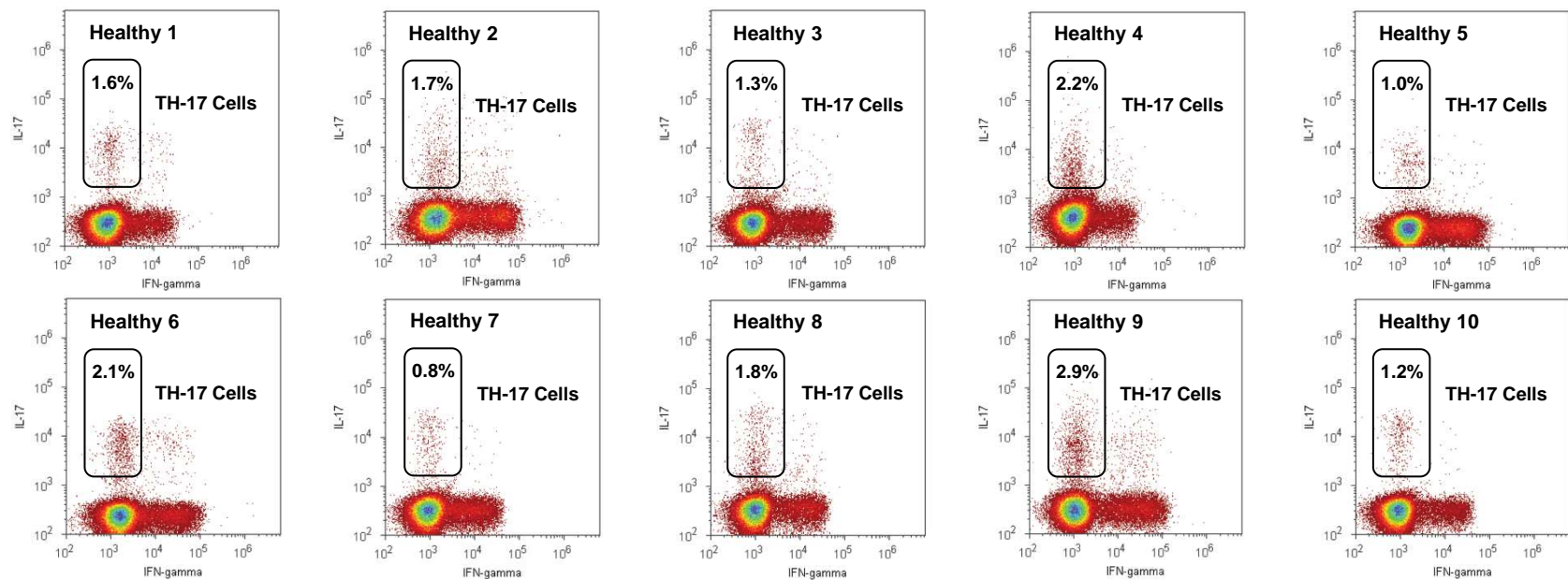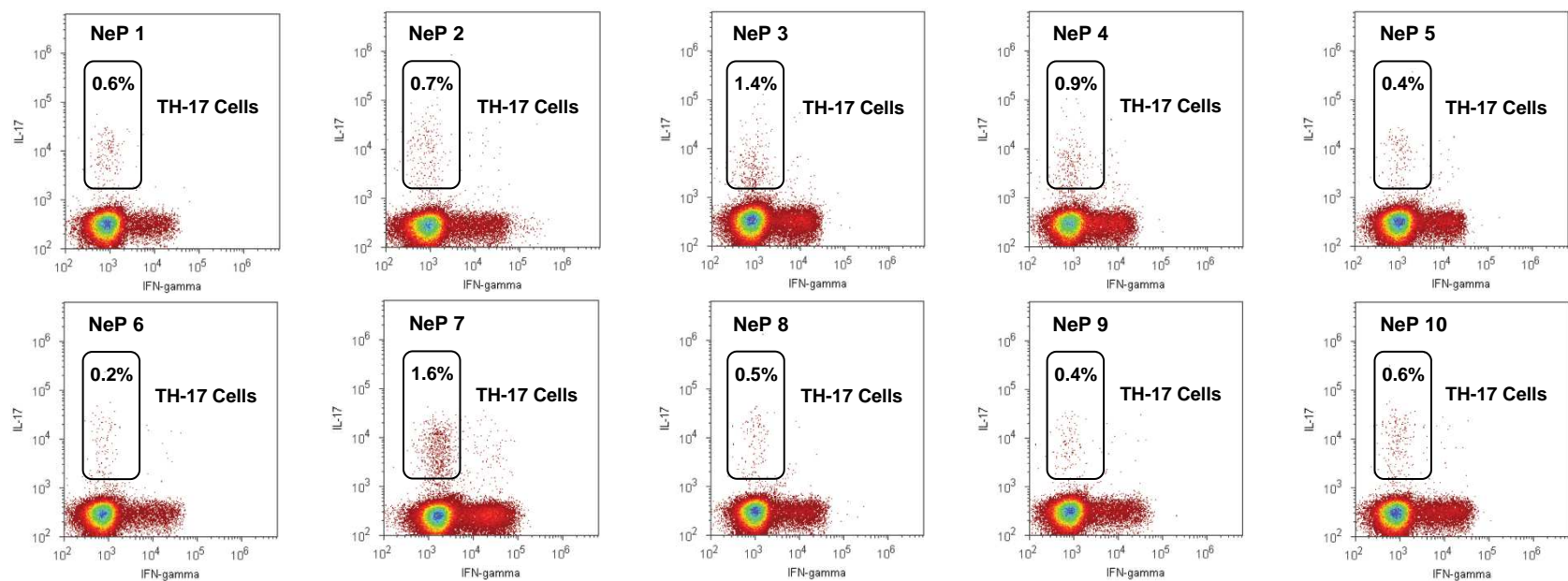

Supplement: Additional file 1: Figure S1. — Exemplary density plots of 10 healthy controls and 10 patients with neuropathic pain showing pro-inflammatory TH17 cells. [file 12974_2014_225_MOESM1_ESM.pdf]

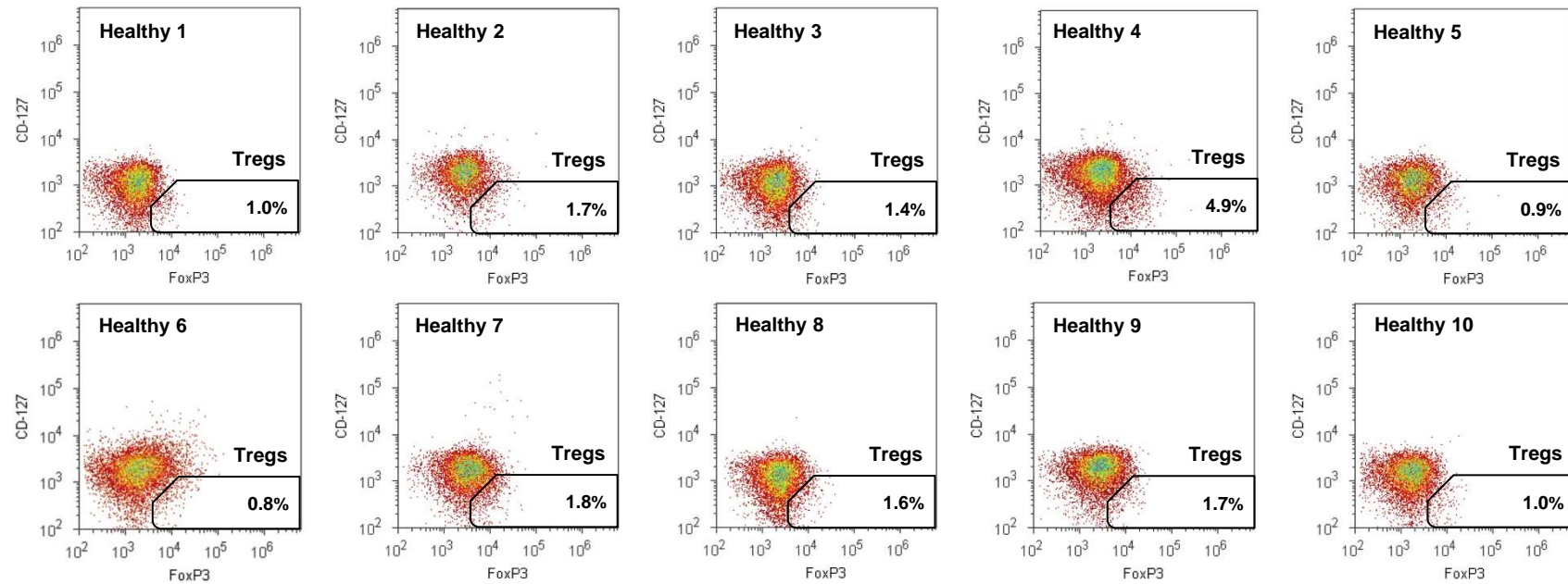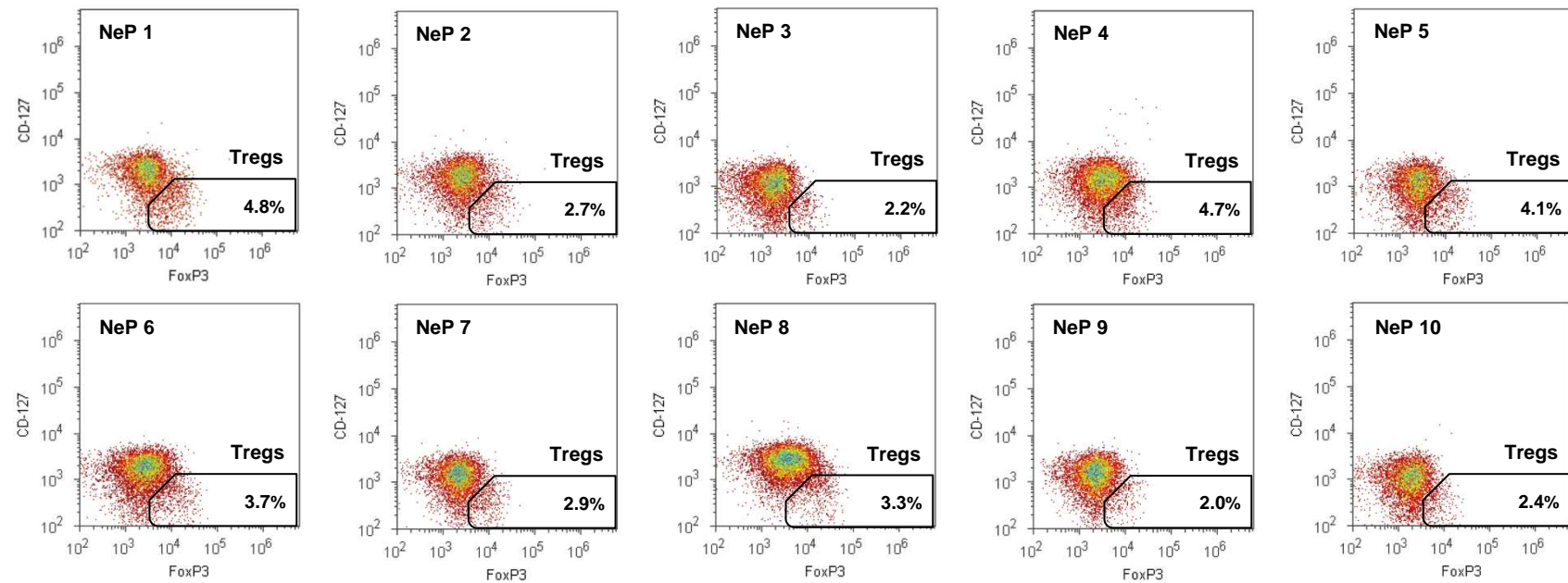

Supplement: Additional file 2: Figure S2. — Exemplary density plots of 10 healthy controls and 10 patients with neuropathic pain showing anti-inflammatory Tregs. [file 12974_2014_225_MOESM2_ESM.pdf]
